# Dasatinib regulates the proliferation and osteogenic differentiation of PDLSCs through Erk and EID3 signals

**DOI:** 10.7150/ijms.87089

**Published:** 2023-09-11

**Authors:** Linglu Jia, Yafei Zhang, Shaoqing Sun, Xingyao Hao, Yong Wen

**Affiliations:** 1 School and Hospital of Stomatology, Cheeloo College of Medicine, Shandong University, Shandong, China.; 2 Shandong Key Laboratory of Oral Tissue Regeneration & Shandong Engineering Laboratory for Dental Materials and Oral Tissue Regeneration, Shandong, China.; 3 Shandong Provincial Clinical Research Center for Oral Diseases, Shandong, China.; 4 Tianjin Stomatological Hospital, School of Medicine, Nankai University, Tianjin, China & Tianjin Key Laboratory of Oral and Maxillofacial Function Reconstruction, Tianjin, China.

**Keywords:** periodontal ligament stem cells, dasatinib, Erk, EID3, osteogenic differentiation, proliferation.

## Abstract

Periodontal ligament stem cells (PDLSCs) are important candidate seed cells for alveolar bone tissue engineering. Dasatinib is a tyrosine kinase inhibitor, and its influence on the osteogenic differentiation of mesenchymal stem cells is a controversial topic. The present study explored the effects of different concentrations of dasatinib on the proliferation and osteogenic differentiation of PDLSCs and tentatively revealed the related mechanism. The results of CCK8 showed that low concentrations of dasatinib (1 nM) did not affect proliferation, while high concentrations of dasatinib significantly inhibited the proliferative activity of PDLSCs. This could be related to the inhibiting effects of dasatinib on Erk signals. ALP staining, alizarin red staining, and western blot proved that low concentrations of dasatinib (1 nM) promoted the osteogenic differentiation of PDLSCs, while high concentrations of dasatinib inhibited it. The negative effects of dasatinib on osteogenic differentiation were reversed when EID3 was knocked down, suggesting that EID3 mediates the regulation of dasatinib on the osteo-differentiation of PDLSCs. Taken together, high concentrations of dasatinib inhibited the proliferation and osteogenic differentiation of PDLSCs through Erk and EID3 signals, while low concentrations of dasatinib could be a potential method to enhance the bone regeneration ability of PDLSCs.

## 1. Introduction

Periodontitis is a common clinical issue in dentistry, while existing clinical treatments have limited effects on the regeneration of defective alveolar bone in periodontitis. Mesenchymal stem cell (MSC)-based bone tissue engineering is a new method to realize alveolar bone regeneration^1^-^3^. As a type of MSC derived from periodontal ligament tissue, periodontal ligament stem cells (PDLSCs), which have good proliferation and osteogenic differentiation capabilities, have been considered candidate seed cells for alveolar bone tissue engineering^4^-^6^. However, the current results of alveolar bone tissue engineering cannot fully meet clinical requirements, because multiple factors such as long-term cultivation^7^ and oxidative stress^8^ could lead to a decrease in the proliferation or multi-differentiation of MSCs. Therefore, it is necessary to find new methods to enhance the tissue regeneration ability of PDLSCs.

Phosphorylation modification of proteins based on tyrosine kinase plays a crucial role in the response to stimuli and the regulation of biological processes in cell 9-11. In MSC, the proliferation and multi-differentiation of cells are also significantly influenced by the activity of tyrosine kinase, so strategies to enhance the regeneration ability of MSC through small molecule preparations targeting tyrosine kinases are receiving increasing attention^12^, ^13^.

Dasatinib, a powerful selective tyrosine kinase inhibitor, has been widely used in clinical practice to treat several types of cancers^14^, ^15^. Interestingly, dasatinib has also been found to regulate the osteogenic differentiation of MSCs, but the research conclusions are controversial. Several studies proved that dasatinib or the combination of dasatinib and quercetin could enhance osteogenic differentiation of MSCs in certain concentration ranges^16^-^20^ and accelerate fracture repair^21^. However, other researchers reported that dasatinib inhibited osteogenic differentiation while promoting adipogenic^22^ or chondrogenic differentiation^23^ in MSCs. Thus, the exact role of dasatinib in the cellular behaviors of MSCs is still unclear, and whether dasatinib can be applied to enhance PDLSC-based alveolar bone regeneration is unknown.

This study explored the influence of dasatinib on the proliferation and osteogenic differentiation ability of PDLSCs and preliminarily revealed the mechanism of dasatinib that was related to Erk and EID3. The results will provide a reference for the potential application of dasatinib in PDLSC-based alveolar bone regeneration.

## 2. Materials and methods

### 2.1 Cell culture

Volunteers without systemic diseases aged 16-24 were included in this study. After obtaining informed consent, we collected premolars or third molars extracted for orthodontic reasons from the volunteers. The teeth were transported to the laboratory, and the periodontal ligament tissues on the middle 1/3 of the root surface were scraped in a sterile environment. The tissues were cut into small pieces, attached to the bottom of a culture bottle and cultured in αMEM medium (Basalmedia, China) containing 10% FBS (IONSERA, Uruguay) at 37 °C in a 5%CO_2_ cell incubator. After the cells migrated from the tissue and grew in the culture flask, the culture medium was exchanged every 2 days. When reaching a confluence of 80%, the cells were digested for passage.

### 2.2 Immunophenotype assay

A BD Stemflow™ hMSC Analysis Kit (BD, USA) was used to incubate PDLSCs with antibodies against CD31, CD34, CD45, CD29, CD73, and CD90 according to the manufacturer's instructions. Then, the immunophenotype of PDLSCs was analyzed by flow cytometry.

### 2.3 Colony forming ability assay

A total of 1000 PDLSCs were cultured in a 10 cm-diameter culture dish. After 2 weeks, the cells were stained with 0.1% crystal violet solution (Solarbio, China) and observed under a microscope. All aggregates comprising more than 50 cells were considered colonies.

### 2.4 Adipogenic induction and Oil Red O staining

Adipogenic medium containing αMEM, 10% FBS, 0.5 mM 3-isobutyl-1-methylxanthine, 10 μg/ml insulin, 1 μM dexamethasone and 0.2 mM indomethacin (Solarbio) was prepared. PDLSCs were cultured in adipogenic medium for 28 days, and the medium was refreshed every 2 days. Finally, the cells were fixed with 4% paraformaldehyde and stained with oil red O (Solarbio) according to the manufacturer's instructions.

### 2.5 Drug application

Dasatinib (Sigma, USA) was dissolved in dimethyl sulfoxide (Solarbio), and then the dasatinib solution was added to the culture medium to make solutions of different concentrations (1, 5, 10, 50, 100, 500, and 1000 nM) as needed. When necessary, an equal amount of dimethyl sulfoxide was added to the medium in the control group. The cells treated with different concentrations of dasatinib were then applied for morphological observation, CCK-8 assay, and western blot.

### 2.6 Osteogenic induction

Osteogenic medium containing αMEM, 10% FBS, 10 nM dexamethasone, 10 mM β-glycerophosphate, and 50 mg/L ascorbic acid (Solarbio) was prepared. Dasatinib was added to the osteogenic medium to achieve concentrations of 1, 2, 5, 10, 20, 50, and 100 nM when necessary. PDLSCs were cultured in osteogenic medium for 4, 7, and 14 days, and the medium was refreshed every 2 days. Then, alkaline phosphatase (ALP) staining and alizarin red staining were used to analyze the osteogenic differentiation of PDLSCs, and western blot and qRT-PCR were used to detect the expression of osteogenic differentiation-related genes.

### 2.7 ALP staining

ALP staining was performed on PDLSCs. Briefly, after being fixed with 4% paraformaldehyde, PDLSCs were stained with an NBT/BCIP staining kit (Beyotime, China) according to the manufacturer's instructions.

### 2.8 Alizarin red staining

PDLSCs were fixed with 4% paraformaldehyde and then stained with alizarin red dye solution (Beyotime) according to the manufacturer's instructions. The mineralized nodules were stained in red, and were observed under a microscope qualitatively.

### 2.9 CCK-8 assay

Cell proliferation activity was detected using a Cell Counting Kit-8 (Dojindo, Kumamoto, Japan). Briefly, PDLSCs were cultured in 96-wellculture plates (1.5×10^3^/ well), and the culture medium contained different concentrations of dasatinib (1, 5, 10, 50, 100, 500, and 1000nM). On day 1, 3, 5, and 7, the culture medium was removed, and then the cells were incubated with 10% CCK-8 solution for 2 hours. The absorbance of the cell solution at 450 nm was measured by a spectrometer.

### 2.10 Total protein isolation and western blot

PDLSCs were washed with cold phosphate buffer solution (PBS) and then incubated in RIPA buffer containing proteinase inhibitor and phosphatase inhibitor (Beyotime) on ice. After ultrasonic lysis and centrifugation, protein solutions were collected, and the concentrations of protein were detected by a BCA assay kit (Biotime). All protein samples were separated by SDS-PAGE gels and transferred to PVDF membranes (Millipore, USA). After being blocked with 5% nonfat-dried milk solution (Beyotime) for 1 hour, the membrane was incubated with primary antibodies overnight at 4 °C, including Erk (#4695, CST, USA), phosphorylated-Erk (p-Erk, #9101, CST), RUNX2 (#8486, CST), COL1 (#39952, CST), EID3 (ab124447, Abcam, UK), GAPDH (abs132004, Absin Bioscience, China), and β-actin (BM3873, Boster, China). Then, the membrane was incubated with secondary antibodies for 1 hour at room temperature. Finally, the protein bands on the membrane were detected by enhanced chemiluminescence (Millipore, Billerica, MA, USA) under an Amersham Imager 600.

### 2.11 Total RNA isolation and qRT-PCR

PDLSCs were washed with cold phosphate buffer solution (PBS), and then total RNA was extracted by RNAios Plus reagent (Takara, Japan) following the manufacturer's instructions. Then, the total RNA was reverse transcribed to cDNA using the PrimeScript™ RT reagent Kit with gDNA Eraser (Takara). qRT-PCR was carried out using a SYBR Green q-PCR kit (Accurate Biology, China) according to the manufacturer's instructions. Finally, the 2^-ΔΔ (ct)^ method was used to compare the values of different groups. The primer sequences are shown in Table 1.

### 2.12 Lentivirus packaging and cell infection

The short hairpin RNA (shRNA) duplex oligo targeting EID3 (shEID3) and a negative control sequence (shNC) were packaged into lentiviruses. PDLSCs were incubated in lentivirus solution supplemented with 8 µg/mL polybrene for 6 hours, and then screened in culture medium containing puromycin for 7 days. Western blot and qRT-PCR were performed to analyze the EID3 knockdown efficiency.

### 2.13 Statistical analysis

All the experiments were repeated at least three times. All data are presented as the mean ± standard deviation (SD). Student's t test was used for comparisons between two groups, and the assessment of variance was used for comparisons among three or more groups, and *p* < 0.05 was considered statistically significant.

## 3. Results

### 3.1 Characterization of PDLSCs

**Figure 1:**
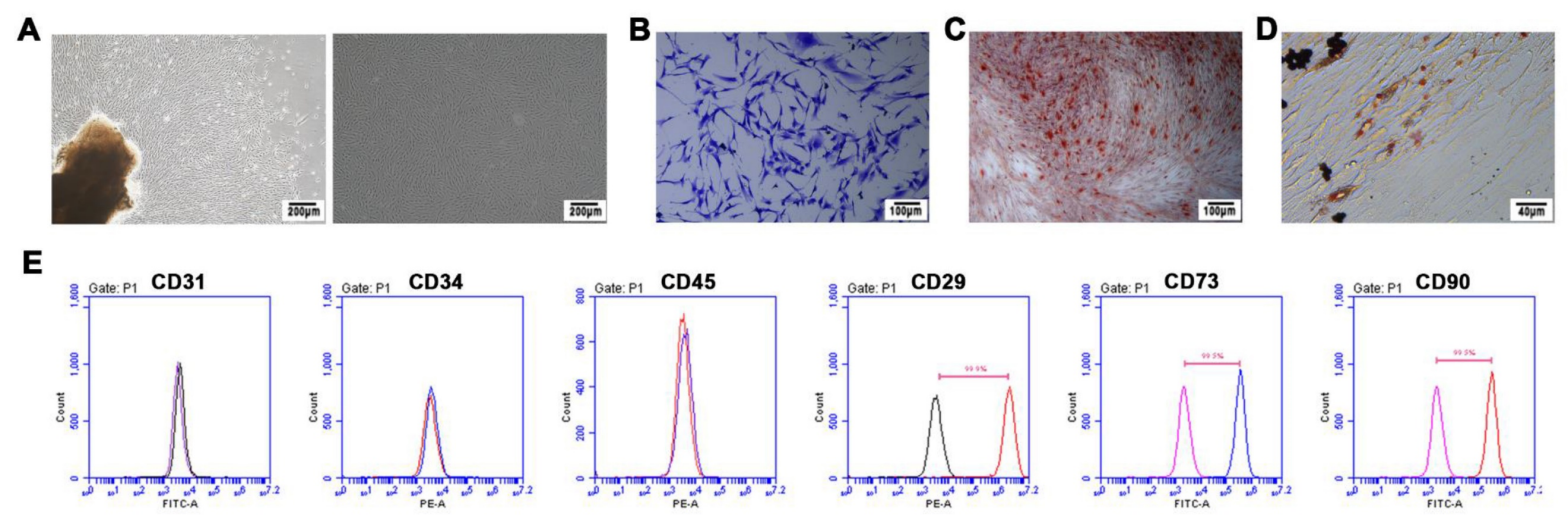
** The culture and identification of PDLSCs. (A)** PDLSCs were in a long spindle shape under the microscope. **(B)** Colony‑forming ability assay of PDLSCs. **(C)** Alizarin red staining of PDLSCs after osteogenic induction, and the mineralized nodules were stained in red.** (D)** Oil red O staining of PDLSCs after adipogenic induction, and the lipid droplets were stained in red. **(E)** The immunophenotype assay of PDLSCs, and PDLSCs were positive for mesenchymal stem cell surface markers CD29, CD73, and CD90, and were negative for hematopoietic or endothelial cell surface markers CD31, CD34, and CD45.

PDLSCs were isolated and cultured successfully. As shown in Figure 1, PDLSCs adhered to the wall of the culture dish and were in a short spindle shape (Figure 1A). Flow cytometry analysis proved that PDLSCs were positive for mesenchymal stem cell surface markers such as CD29, CD73, and CD90 and were negative for hematopoietic or endothelial cell surface markers such as CD34, CD45, and CD31 (Figure 1E). In the colony forming assay, a single cell formed clones of more than 50 cells, indicating that PDLSCs have a strong self-renewal ability (Figure 1B). Alizarin red staining (Figure 1C) and oil red O staining (Figure 1D) separately proved that PDLSCs could synthesize mineralized nodules or lipid droplets after osteogenic or adipogenic induction, proving that PDLSCs had multidirectional differentiation ability.

### 3.2 The influence of dasatinib on PDLSC proliferation

**Figure 2:**
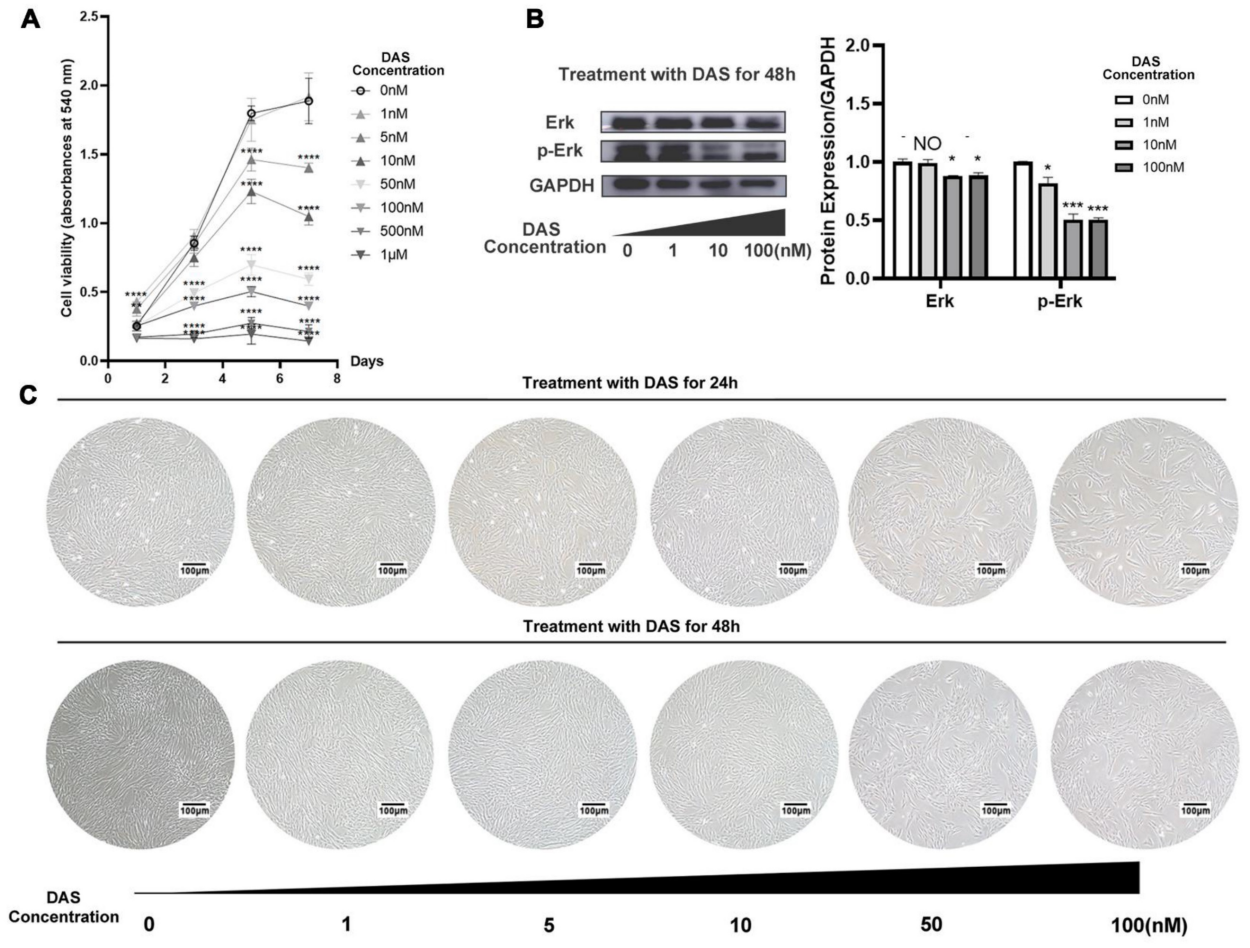
** The influence of dasatinib on PDLSC proliferation. (A)** CCK-8 assay of PDLSCs when the cells were treated with different concentrations of dasatinib for 7 days. **(B)**The protein level of Erk and p-Erk were analyzed by western blot when PDLSCs were treated with different concentrations of dasatinib for 48 hours. **(C)** Morphology of PDLSCs under the microscope when the cells were treated with different concentrations of dasatinib for 24 and 48 hours. DAS: dasatinib. NO: *p*≥0.05. *: *p*<0.05. **: *p*<0.01. ***: *p*<0.001. ****: *p*<0.0001.

After different concentrations of dasatinib were added to the culture medium, the proliferative activity of PDLSCs was detected by CCK-8. The results showed that 1 nM dasatinib did not have a significant influence on the proliferation of PDLSCs within 7 days (Figure 2A). However, when the concentration increased to 5, 10, 50, 100, 500, and 1000 nM, dasatinib exhibited an inhibitory effect on proliferative activity as the concentration increased (Figure 2A). We also compared the cell morphology of PDLSCs when they were incubated with different concentrations of dasatinib. As shown in Figure 2C, a high concentration of dasatinib caused the cell morphology to become short and hypertrophic and the number of cells to decrease. Therefore, low concentrations of dasatinib (1 nM) had no effects on PDLSC proliferation, while high concentrations of dasatinib (5 to 1000 nM) inhibited proliferative activity.

### 3.3 The influence of dasatinib on the Erk signaling pathway

Erk signaling pathways have been reported to positively regulate the proliferation of stem cells, so the influence of dasatinib on the expression of Erk and p-Erk was analyzed. The western blot results showed that high concentrations of dasatinib (10 and 100 nM) significantly inhibited the phosphorylation of Erk (Figure 2B), which may be related to the inhibition of cell proliferation by dasatinib.

### 3.4 The influence of dasatinib on PDLSC osteogenic differentiation

**Figure 3:**
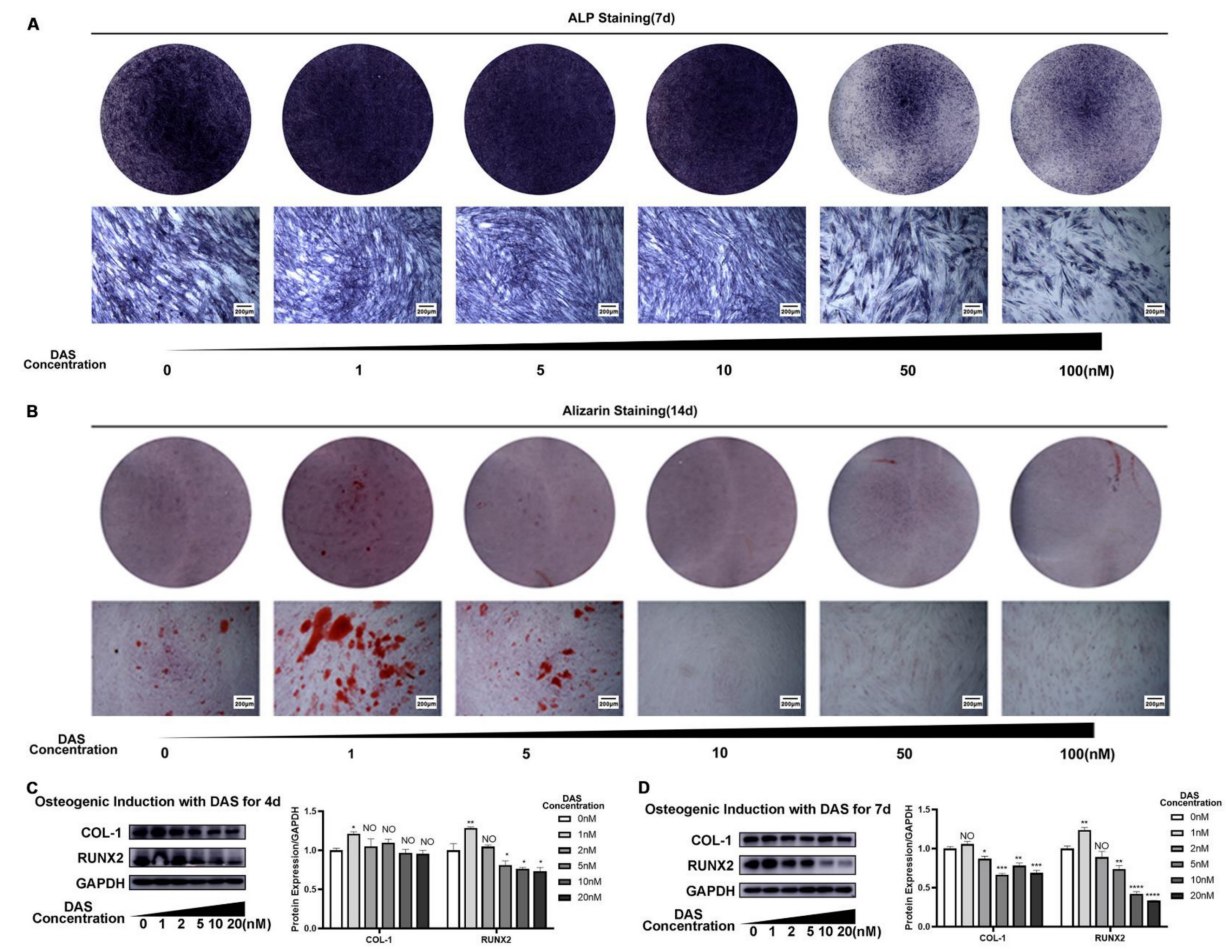
** The influence of dasatinib on PDLSC osteogenic differentiation. (A)** ALP staining after PDLSCs were treated with osteogenic induction medium containing different concentrations of dasatinib for 7 days. **(B)** Alizarin red staining after PDLSCs were treated with osteogenic induction medium containing different concentrations of dasatinib for 14 days. **(C)** The expressions of COL1 and RUNX2 were analyzed by western blot after PDLSCs were treated with osteogenic induction medium containing different concentrations of dasatinib for 4 days. **(D)** The expressions of COL1 and RUNX2 were analyzed by western blot after PDLSCs were treated with osteogenic induction medium containing different concentrations of dasatinib for 7 days. DAS: dasatinib. NO: *p*≥0.05. *: *p*<0.05. **: *p*<0.01. ***: *p*<0.001. ****: *p*<0.0001.

After dasatinib was added to the osteogenic induction medium, the osteogenic differentiation ability of PDLSCs was analyzed. ALP staining on Day 7 was stronger in the 1, 5 and 10 nM dasatinib groups than in the control group, while the 50 and 100 nM dasatinib groups had weaker ALP staining than the control group (Figure 3A). Similarly, the results of alizarin red staining on Day 14 showed that 1 and 5 nM dasatinib increased the number of mineralized nodules, while dasatinib at concentrations higher than 10 nM decreased the number of mineralized nodules (Figure 3B). We also analyzed the expression of osteogenic differentiation-related genes, and the results showed that low concentrations of dasatinib (1 nM) increased the protein levels of COL-1 and RUNX2, while high concentrations of dasatinib (5, 10 and 20 nM) inhibited their expression (Figure 3C, D) on Day 7. All of the above results showed that low concentrations of dasatinib (1nM) promoted the osteogenic differentiation of PDLSCs, while high concentrations of dasatinib inhibited it.

### 3.5 Dasatinib inhibits the osteogenic differentiation of PDLSCs through EID3

**Figure 4:**
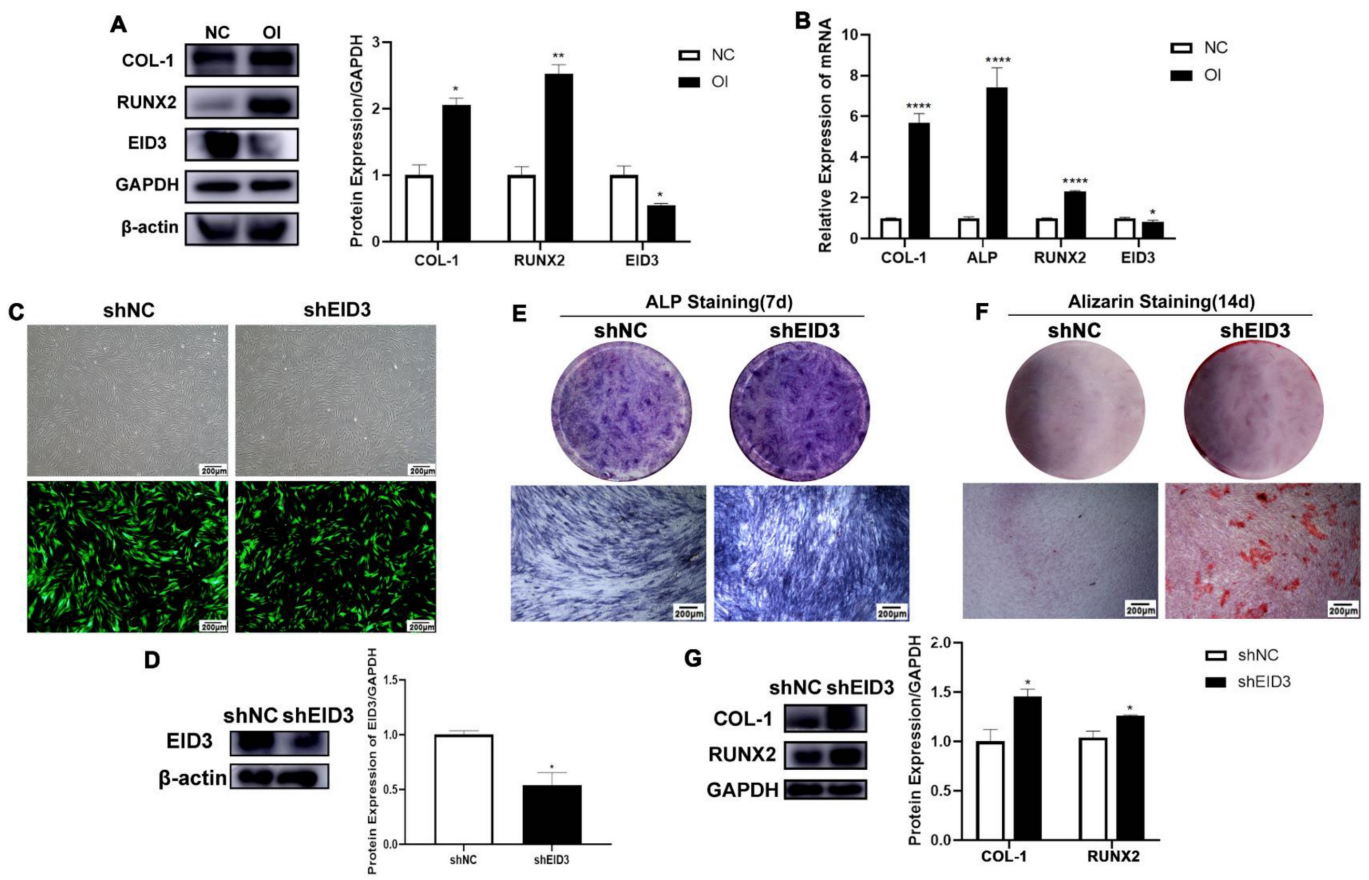
** EID3 inhibits the osteogenic differentiation of PDLSCs. (A)** The protein levels of EID3, COL1, and RUNX2 were analyzed by western blot when PDLSCs were osteogenic induced for 7 days. **(B)** The mRNA levels of EID3, COL1, and RUNX2 were analyzed by qRT-PCR when PDLSCs were osteogenic induced for 7 days. **(C)** PDLSCs infected with lentivirus showed green fluorescence under fluorescence microscope. **(D)** The efficiency of knockdown of EID3 by lentivirus was analyzed by western blot. **(E)** ALP staining of PDLSCs when they were osteogenic induced for 7 days. **(F)** Alizarin red staining of PDLSCs when they were osteogenic induced for 14 days. **(G)**The expressions of COL1 and RUNX2 were analyzed by western blot when PDLSCs were osteogenic induced for 7 days. NC: cells cultured in culture medium. OI: cells cultured in osteogenic induction medium. shEID3: PDLSCs with EID3 knocked down. shNC: the control group for shEID3 PDLSCs. *: *p*<0.05. **: *p*<0.01. ***: *p*<0.001. ****: *p*<0.0001.

EID3 has been reported to be an inhibitor of cell differentiation. To confirm the role of EID3 in PDLSCs, we first detected the expression of EID3 in PDLSCs. The results of western blot and qRT-PCR showed that the mRNA and protein levels of EID3 in osteo-differentiated PDLSCs were lower than those in nondifferentiated cells, suggesting that EID3 was negatively correlated with osteogenic differentiation (Figure 4A, B). Next, lentivirus was used to knock down EID3 in PDLSCs, and the knockdown efficiency was confirmed by western blot (Figure 4C, D). Subsequently, PDLSCs with EID3 knocked down were cultured in osteogenic induction medium. After 7 and 14 days, the shEID3 group had stronger ALP staining (Figure 4E), stronger alizarin red staining (Figure 4F), and higher expression levels of COL-1 and RUNX2 than the shNC group (Figure 4G). All these results proved that EID3 was a negative regulator of osteogenic differentiation in PDLSCs.

**Figure 5:**
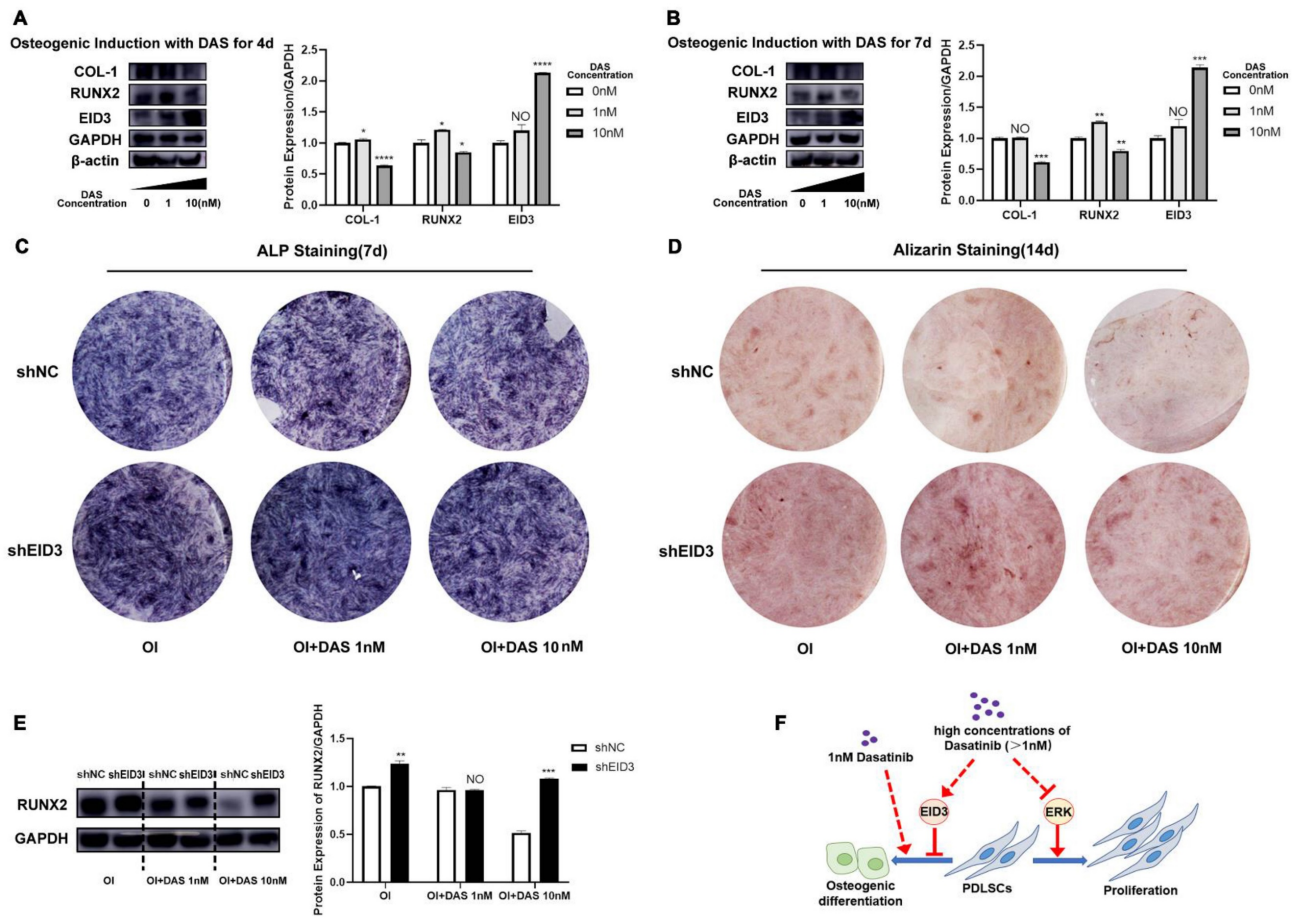
** Dasatinib inhibits the osteogenic differentiation of PDLSCs through EID3. (A) (B)**The expressions of EID3, COL1, and RUNX2 were analyzed by western blot when PDLSCs were treated with osteogenic induction medium containing different concentrations of dasatinib for 4 and 7 days. **(C)** ALP staining when PDLSCs with EID3 knocked down were treated with osteogenic induction medium containing different concentrations of dasatinib. **(D)** Alizarin red staining when PDLSCs with EID3 knocked down were treated with osteogenic induction medium containing different concentrations of dasatinib. **(E)** The protein levels of RUNX2 were analyzed by western blot when PDLSCs with EID3 knocked down were treated with osteogenic induction medium containing different concentrations of dasatinib. **(F)** A summary: high concentration of dasatinib (>1nM) inhibited the proliferation and osteogenic differentiation of PDLSCs through inhibiting the Erk signal and promoting the EID3 signal, while low concentration of dasatinib (1nM) promoted osteogenic differentiation of PDLSCs without affecting proliferation. OI: osteogenic induction. DAS: dasatinib. shEID3: PDLSCs with EID3 knocked down. shNC: the control group for shEID3 PDLSCs. NO: *p*≥0.05. *: *p*<0.05. **: *p*<0.01. ***: *p*<0.001. ****: *p*<0.0001.

When PDLSCs were treated with 10 nM dasatinib during osteogenic induction, the expression of EID3 increased significantly (Figure 5A, B), so we speculated that the effects of dasatinib on PDLSC osteogenic differentiation were related to EID3. To confirm this hypothesis, we knocked down EID3 in PDLSCs treated with 10 nM dasatinib. As shown in Figure 5C, D, and E, the results of ALP staining, Alizarin staining and western blot showed that 10 nM dasatinib inhibited the osteogenic differentiation of PDLSCs, while when EID3 was knocked down, the effects of dasatinib were reversed. These results proved that dasatinib inhibited the osteogenic differentiation of PDLSCs through EID3.

## 4. Discussion

In PDLSC-based alveolar bone tissue engineering, maintaining a high level of proliferative activity of cells is quite important for the regeneration of tissue. The present study proved that low concentrations of dasatinib (1 nM) did not affect PDLSC proliferation, while high concentrations of dasatinib (5-1000 nM) significantly inhibited the proliferative activity of PDLSCs. In addition, as the concentration of dasatinib increased, its inhibitory effect on proliferation became stronger. In fact, previous studies focusing on the anticancer effect of dasatinib have shown that dasatinib can induce cell cycle arrest and apoptosis in cancer cells, so the cancer cells treated with dasatinib showed decreased proliferative activity^24^, ^25^. For MSCs, dasatinib at 5-50 nM has been reported to reduce the number of cell divisions in MSCs and osteoprogenitor cells 16, 18, which is in line with our study. Therefore, it is recommended to use low doses of dasatinib in the study of PDLSC-based tissue regeneration to maintain the proliferative activity of cells.

The mitogen-activated protein kinase (MAPK) signaling pathway is a classic signaling cascade pathway that can transmit extracellular stimuli into the cell and participate in the regulation of cell proliferation, differentiation, apoptosis and so on^26^, ^27^. Our previous studies have also proven that the MAPK/Erk pathway is a positive regulator of the proliferation of PDLSCs^28^. The present results showed that a high concentration of dasatinib significantly inhibited the phosphorylation of Erk, which may be one of the mechanisms by which dasatinib inhibits the proliferative activity of PDLSCs. Similarly, several studies have revealed the inhibitory effects of dasatinib on the MAPK/Erk pathway in some types of cancer cells and MSCs^18^, ^29^-^31^. However, single use of dasatinib was also reported to promote the activation of MAPK/Erk signaling pathways in melanocytes^32^, or have no effects on MAPK/Erk in nonclear cell renal cell carcinoma^33^. These inconsistent results suggest that the effects of dasatinib may be diverse in different types of cells, and its influence and mechanism in PDLSCs needs to be further explored.

The influence of dasatinib on the osteogenic differentiation ability of PDLSCs is another aspect to consider. The present results showed that the effect of dasatinib varied with the change in drug concentration: treatment with a low concentration of dasatinib enhanced the osteo-differentiation ability of PDLSCs, while a high concentration of dasatinib led to a decrease in osteogenic differentiation potential. In fact, previous studies on the relationship between dasatinib and the osteogenic differentiation of MSCs are also controversial: several studies provided evidence on the positive effects of dasatinib on MSC osteo-differentiation^16^-^20^, while others supported that dasatinib inhibited the tendency of MSCs to differentiate toward bone^22^, ^23^. This may be related to multiple factors, such as the concentration of dasatinib, the duration of treatment, and the source of MSCs. Therefore, it is important to control the drug concentration during the application of dasatinib, and we recommend using 1 nM dasatinib to promote the osteogenic differentiation of PDLSCs without inhibiting proliferation. One of the limitations of the present study is that only *in vitro* cell experiments were conducted, and animal and preclinical experiments are still needed to further verify the positive effect of dasatinib on bone tissue regeneration, which will be the focus of our future research. Fortunately, considering that dasatinib has been clinically used in the treatment of leukemia 14, 15, it may be easier to obtain acceptance and promotion when using dasatinib in promoting PDLSC-based alveolar bone regeneration in clinical practice.

Exploring the mechanism by which dasatinib regulates the osteodifferentiation of PDLSCs can help to identify molecular targets for enhancing PDLSC-based bone regeneration. Our results showed that a high concentration of dasatinib significantly improved the expression of EID3. EID3 is a member of the EID (E1A-like inhibitor of differentiation) family, which has been proven to affect DNA methylation and gene transcription and thus regulates cell functions^34^, ^35^. Several studies have revealed that EID3 is a negative regulator of the differentiation of MSCs^34^, ^36^, ^37^. The present study proved that knocking down EID3 partly reversed the inhibitory effects of dasatinib on the osteogenic differentiation of PDLSCs, suggesting that the regulation of high concentrations of dasatinib on PDLSCs is related to EID3. Currently, the molecular mechanism by which dasatinib regulates MSC differentiation has not been fully elucidated. A study focusing on chondrogenic differentiation proved that dasatinib regulated the transduction of the Hippo-YAP signaling pathway in MSCs^23^. Other studies suggested that dasatinib affected the activation of BMP 17 and β-catenin signaling 18. Our study proposes a regulatory relationship between dasatinib and EID3, providing a new idea for the study of the mechanism of dasatinib. We will explore the molecular transduction mechanism of dasatinib in inhibiting tyrosine kinase and regulating EID3 expression in our future studies.

## 5. Conclusion

The present study revealed that a high concentration of dasatinib inhibited the proliferation and osteogenic differentiation of PDLSCs through Erk and EID3 signals, while a low concentration of dasatinib promoted osteogenic differentiation without affecting proliferation (Figure 5F). Therefore, 1 nM dasatinib is suggested to be applied to enhance PDLSC-based alveolar bone tissue engineering. These results provide a scientific basis and reference for the clinical translation of dasatinib in alveolar bone regeneration in the future.

## Figures and Tables

**Table 1:** The sequences of primers.

GENE	Forward primer 5'-3'	Reverse primer 5'-3'
COL1	GCTGATGATGCCAATGTA	CCAGTCAGAGTGGCACAT
RUNX2	AGGCAGTTCCCAAGCATT	TGGCAGGTAGGTGTGGTA
EID3	CTCACCGCTGACGAGGAG	CTTCTCTGGTTCGGCTCA
GAPDH	AGAAGGCTGGGGCTGAGA	AGGGGCCATCCACAGTCT
